# The common incidence-age multistep model of neurodegenerative diseases revisited: wider general age range of incidence corresponds to fewer disease steps

**DOI:** 10.1186/s13578-021-00737-8

**Published:** 2022-01-29

**Authors:** Daniela Gerovska, Marcos J. Araúzo-Bravo

**Affiliations:** 1 Computational Biology and Systems Biomedicine, Biodonostia Health Research Institute, Calle Doctor Begiristain s/n, 20014 San Sebastian, Spain; 2 Computational Biomedicine Data Analysis Platform, Biodonostia Health Research Institute, Calle Doctor Begiristain s/n, 20014 San Sebastian, Spain; 3 Basque Foundation for Science, IKERBASQUE, Calle María Díaz Harokoa 3, 48013 Bilbao, Spain; 4 CIBER of Frailty and Healthy Aging (CIBERfes), Madrid, Spain; 5 Max Planck Institute for Molecular Biomedicine, Computational Biology and Bioinformatics Group, Röntgenstr. 20, 48149 Münster, Germany

**Keywords:** Multistep model, Neurodegenerative diseases, Integration of epidemiological data, Incidence-age, Comprehensive model

## Abstract

**Background:**

Previously, we collected age-stratified incidence data of 404 epidemiological datasets of 10 neurodegenerative diseases (NDs), namely Amyotrophic Lateral Sclerosis (ALS), Alzheimer’s disease (AD), Parkinson’s disease (PD), Huntington’s disease (HD), Fronto Temporal Dementia (FTD), Dementia with Lewy Bodies (DLB), Parkinsonism (PDM), Parkinson’s disease with Dementia (PDD), Creutzfeldt–Jakob disease (CJD), and Multiple Sclerosis (MS). We tested whether each ND follows a multistep model, found the number of steps necessary for the onset of each ND, found the number of common steps with other NDs and the number of specific steps of each ND, and built a parsimony tree of the genealogy of the NDs. The tree disclosed three groups of NDs: the stem NDs with less than 3 steps; the trunk NDs with 5–7 steps; and the crown NDs with more than 7 steps.

**Methods:**

We made a multidimensional reduction of the previously collected age-stratified incidence epidemiological data of the 10 NDs. We studied the general range of incidence of the 10 NDs using the age- and sex-stratified incidence data. First, we calculated the log of the incidence versus the log of the age for each ND. Next, we calculated the age intervals of the spread of the incidence of each ND. We calculated the regression of the steps obtained with the multistep model versus the age of incidence of the NDs.

**Results:**

We found that the number of steps of the NDs is inversely correlated with the age of incidence of the NDs, and calculated the number of years required for a single step for each ND. Based on these results, we extended the genealogy tree model of the NDs to account for the time needed for a ND step to occur.

**Conclusion:**

The extended genealogy tree disclosed three groups of NDs according to the estimated time needed for a step to occur: the stem ND, HD, with 32.5 years/step, the trunk NDs ALS, FTD, PD and CJD, with 6.7–13.7 years/step; and the crown NDs PDM, PDD, AD and DLB, with 2.3–3.8 years/step. Thus, the NDs cluster into three groups according to both the number of steps and the number of years for a step to occur.

## Introduction

Neurodegenerative diseases (NDs) are characterized with progressive loss of cognitive and/or motor function. Human genetics studies have shown that disease-causing rare mutations and risk-associated common alleles overlap in different neurodegenerative disorders [1]. Intricate genotype–phenotype relationships and common cellular pathways emerged from recent genetic and mechanistic studies [1, 2]. Shared pathological mechanisms include defective protein quality-control and degradation pathways, dysfunctional mitochondrial homeostasis, stress granules, and maladaptive innate immune responses [1]. Accumulation of misfolded proteins is shared among NDs [3–5]. Both malignant transformation and neurodegeneration are complex and lengthy multistep processes characterized by abnormal expression, post-translational modification, and processing of certain proteins. To maintain and allow the accumulation of these dysregulated processes, and to facilitate the step-wise evolution of the disease phenotype, cells co-opt a compensatory regulatory mechanism, with this role attributed to Hsp90 in cancer and proposed to have a similar role in neurodegeneration [6].

Many researchers [2, 5, 7–12] used a model [7] originally applied to cancer epidemiology to investigate the hypothesis that certain NDs are multistep processes based on incidence—age data and found that specific NDs are consistent with a multistage model of the respective disease in a certain population pool. Estimating the slope *m *of the linear model, they identified *n*  =  *m* + 1 steps of the disease process and looked to the identification of these steps that could lead to preventive and therapeutic avenues.

Al-Chalabi et al. [2] first applied the multistep model used in cancer epidemiology on the similarly developing Amyotrophic Lateral Sclerosis (ALS), and found an overall slope of 4.8, 4.6 for men, and 5.0 for women, when looking for a linear relationship between log(incidence) and log(age) in five registers from a catchment population of about 34 million people. The slope estimate suggested that ALS is a six-step process with six factors involved in the disease onset [2]. The factors remain unidentified; anyway it has been found that fewer steps are predicted for those carrying a known ALS-causing mutation [8]. Vucic et al. [11] suggested that six steps were required in Japanese and Australian patients with ALS while 5 steps were needed in South Korean patients. Garton et al. [9] tested whether men with a psychiatric disorder or cardiovascular disease (CVD) diagnosis who have an increased relative risk of ALS would have decreased the predicted steps to disease. They found that for the general Danish population the regression coefficient was 4.6, i.e. six steps and this did not differ when considering ALS cases with a prior psychiatric but surprisingly, it was higher, seven steps, for those with a prior CVD diagnosis. Assessing sex differences, Garton et al. [9] data and analyses suggested half a step fewer for men without support for contributing differences explained by menopause.

The age-specific incidence of Parkinson’s disease (PD) is also consistent with a process that develops in multiple, discrete steps [5], six for both men and women. Le Heron et al. specified that this number is on average six before age 45 and eight after [12].

Gerovska et al. [5] identified 11 steps in men and 13 steps in women in Alzheimer’s disease (AD). Licher et al. [10] found that AD required 14 steps before disease manifestation, suggesting that genetically predisposed individuals require fewer steps indicating that they already inherited multiple of these steps.

Additionally, Gerovska et al. [5] identified the necessary number of steps in Huntington’s disease (HD), 2 and 2, Dementia with Lewy Bodies (DLB), 13 and 12, Parkinsonism (PDM), 8 and 9, Parkinson’s disease with Dementia (PDD), 11 and 9, and Creutzfeldt–Jakob disease (CJD), 6 and 6, for men and women, separately. Due to the few epidemiological data available, the Fronto Temporal Dementia (FTD) multistep model was applied on combined male and female data and identified six steps [5].

The common incidence-age multistep model, presented as a genealogy tree of the NDs, accounts for shared steps required for the onset of the specific diseases [5]. Alongside the number of steps necessary for a ND to occur, the common steps are represented by the trunk of the tree, and the non-common, specific steps by the branches of the tree. The tree disclosed three types of NDs: the stem NDs with less than 3 steps; the trunk NDs with 5–7 steps; and the crown NDs with more than 7 steps. The tree has three levels: The stem proximal level with a non-step disease like MS, and a purely genetic disease like HD; The middle trunk level with the cluster of ALS, PD, FTD and CJD; and the crown with AD, DLB, and the Parkinson-associated diseases—PDD and PDM. The tree provides a comprehensive understanding of the relationship across the different NDs, as well as a mathematical framework for dynamic adjustment of the genealogical tree of the NDs with the appearance of new data from epidemiological studies and the addition of new NDs to the model [5].

Here we view the general multistep model of the NDs in context of the number of years required for a single step for each ND to occur, and present a new revised genealogy tree of the NDs based on incidence-age epidemiological data taking into account these years per step.

## Materials and methods

### Epidemiological data

Previously we collected 404 datasets on age-stratified incidence of the major NDs: AD, PD, HD, ALS, FTD, as well as DLB, PDM, PDD and CJD, and under the assumption that they share pathogenic mechanisms, we studied whether such mechanisms have left a fingerprint on the dynamics of their incidence patterns with age and whether such fingerprints can provide insights about the ND triggering mechanisms. We used as a control Multiple Sclerosis (MS), a disease with a neurodegenerative component, though not as central as in the diseases mentioned above. A full list of the data sources is given in Table S1 and the reference list of the Additional file Gerovska et al. [5]. To recalculate the model of the genealogy tree based on all data, we excluded 7 total AD datasets from the data in Table S1: AD-61, AD-62 [13], AD-68 [14], AD-71 [15], AD-78 [16], which have male and female counterparts in the data, and AD-60 [17] and AD-82 [18] without counterpart data sets annotated for sex.

### Multistep model

The incidence rate is the number of new cases per population at risk in a given time period. When the denominator is the sum of the person-time of the at risk population, it is also known as the incidence density rate or person-time incidence rate. The prevalence is the proportion of cases in the population at a given time rather than rate of occurrence of new cases. Thus, incidence conveys information about the risk of contracting the disease, whereas prevalence indicates how widespread the disease is. Prevalence is the proportion of the total number of cases to the total population and is more a measure of the burden of the disease on society with no regard to time at risk or when subjects may have been exposed to a possible risk factor.

For a multistep model the incidence *i* across the time is *i*  = *u*_1_⋅*u*_2_⋅…*u*_*n*−1_⋅*u*_*n*_⋅*t*^(*n*−1)^ where *u*_*k*_ is the average background risk of step *k.* Applying the log transform to both sides of the above equation, the regression line in log scale of the incidence *i* across the time *t* is log(*i*)  = (*n* − 1)⋅log(*t*) + *c*, where *m * =  *n* − 1 is the slope of the regression line and *c*  = log(*u*_1_⋅*u*_2_…*u*_*n*−1_⋅*u*_*n*_) = log(*u*) is the intercept. Whereas the background risk *u* of all steps is *u * = e^*c*^, the number of steps *n* is *n*  = *m*  + 1, and the geometric average background risk of all steps μ(*u*) is μ(*u*)  = *u*^(1/n)^.

We have truncated the data corresponding to ages higher or equal than 80 years with the condition of at least 4 data points remaining. The disease names finishing in ‘f’ lowercase correspond to female samples, those finishing in ‘m’ correspond to male samples. Those with all characters in uppercase correspond to the pool of the two sexes.

### Integrative analysis of the trajectories of incidence versus age of the NDs

To adjust the epidemiological data studies to same age intervals, we modeled the age-stratified incidence trajectories of each study with cubic splines and interpolated each trajectory at the same age points for all datasets. We averaged the incidence trajectories of the different studies corresponding to the same ND *i*, and built a size *d*  ×  *a* incidence matrix *I,* where *d* and *a* are the number of NDs and age points, respectively. The element *I*(*i, j*) denotes the incidence of disease *i* at age *j*.

### Calculation of the genealogy tree of the NDs

The mathematical method for the branching of the genealogy tree based on incidence-age data is described in detail in Gerovska et al. [5] and its main steps are illustrated in Fig. 6 for the tree construction based on pooled data of the male, female, and data without annotation for sex. To reduce the search space of common-step combinations, we use a parsimony approach imposing a “preserving the ordinal number of each step” criterion, assuming that the ordinal number of a step in a disease is the same ordinal number of this same step in another disease. Among all potential common steps, we choose those with higher plausibility to be common among more diseases, introducing “maximizing the number of shared steps between diseases” criterion.

### Representation of the extended genealogy tree of the NDs

The tree of the genealogy of the NDs shows the number of steps necessary for a ND to occur. The common steps are represented by the trunk of the tree, and the non-common, specific steps, by the branches of the tree. The left and right tree sides in the sex-stratified model depict the specific branches of the male and female NDs, NDm and NDf, respectively. The width of the trunk in the extended tree model is equal to the number of years for a step to occur for the ND whose specific steps branch out of the common trunk at a point. If more NDs with specific steps branch out of the same trunk point, then the width of the trunk is equal to the mean of the years/step for all these NDs.

## Results

### The multidimensional reduction analysis reveals three categories of NDs

**Fig. 1:**
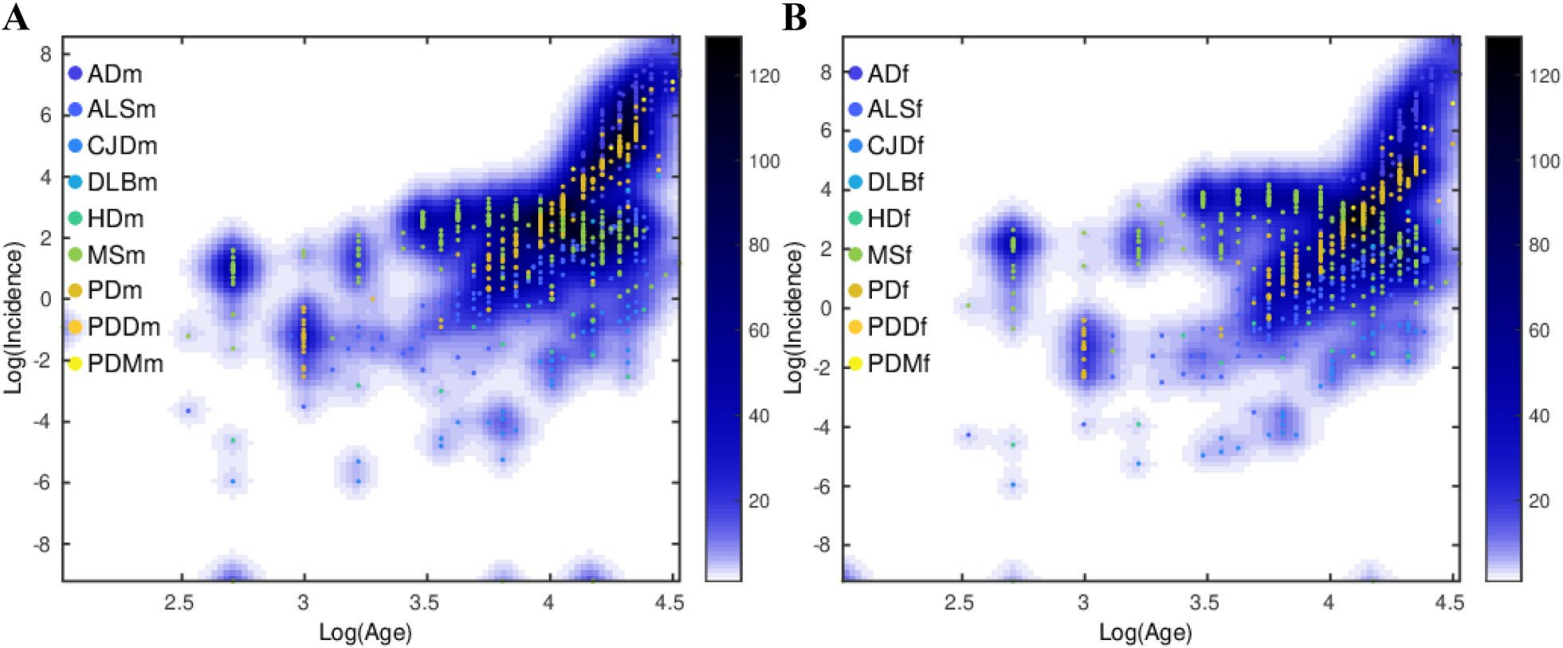
Scatter plots of the log_10_ (incidence) versus the log_10_ (age) for each ND. **A** Male. **B** Female. The color of the density plot is proportional to the frequency of the data points

**Fig. 2:**
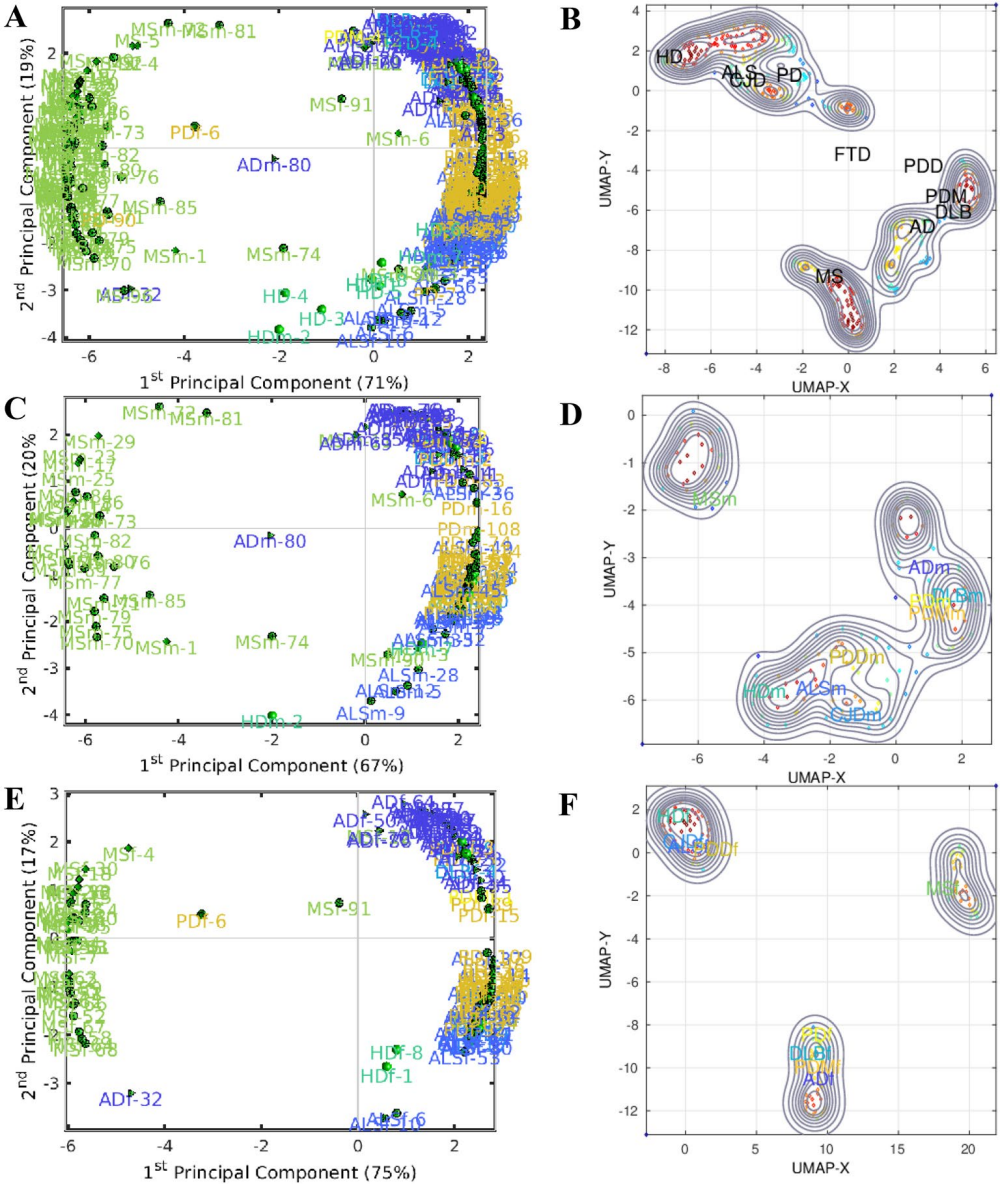
Principal Component Analysis (PCA) for **A** all data **C** male **E** female, and Uniform Manifold Approximation and Projection (UMAP) for **B** all data **D** male **F** female. The numbers next to the disease names in the PCAs correspond to the IDs of the datasets in the exhaustive list from Table S1 in the Additional file in Gerovska et al. [5]

Previously, we have analyzed 404 epidemiological datasets described in detail in the Additional file of Gerovska et al. [5]. Here, we revisit this data and the general multistep model to account for the age range intervals of the specific ND incidence. The number of age intervals with incidence information across the datasets analyzed was 2530; see Fig. 1, which shows the raw incidence-age data and illustrates the monotonous increase of the incidence of NDs with age for both male and female except for MS. We used two methods to make a multidimensional reduction of the age- and sex-stratified incidence epidemiological data of the 10 NDs adjusted to the same age intervals, namely principal component analysis (PCA) and a uniform manifold approximation and projection (UMAP) [19]. UMAP is useful for identifying clusters when the number of clusters is not known in advance and when there is a high number of significant PCs. The first component of the PCA (PC1) explains 71%, 67% and 75% of the variability of the incidence-age data profiles for all, male, and female data, respectively (Fig. 2A, C, E). PC1 separates well the MS data (which does not follow a multistep model and serves as a reference in the genealogy tree model) from all the other multistep NDs. The second component of the PCA (PC2) explains the 19%, 20% and 17% for all, male, and female data. In general, PC2 separates the profiles of the NDs from the middle trunk of the tree (those with fewer steps) from those in the crown of the tree (more steps). The UMAPs (Fig. 2B, D, F) cluster together the incidence-age data according to their belonging to one of the three levels of the genealogy tree, leaving out the MS data cluster.

### Higher incidence age range corresponds to less steps *n* required to trigger the ND

**Fig. 3:**
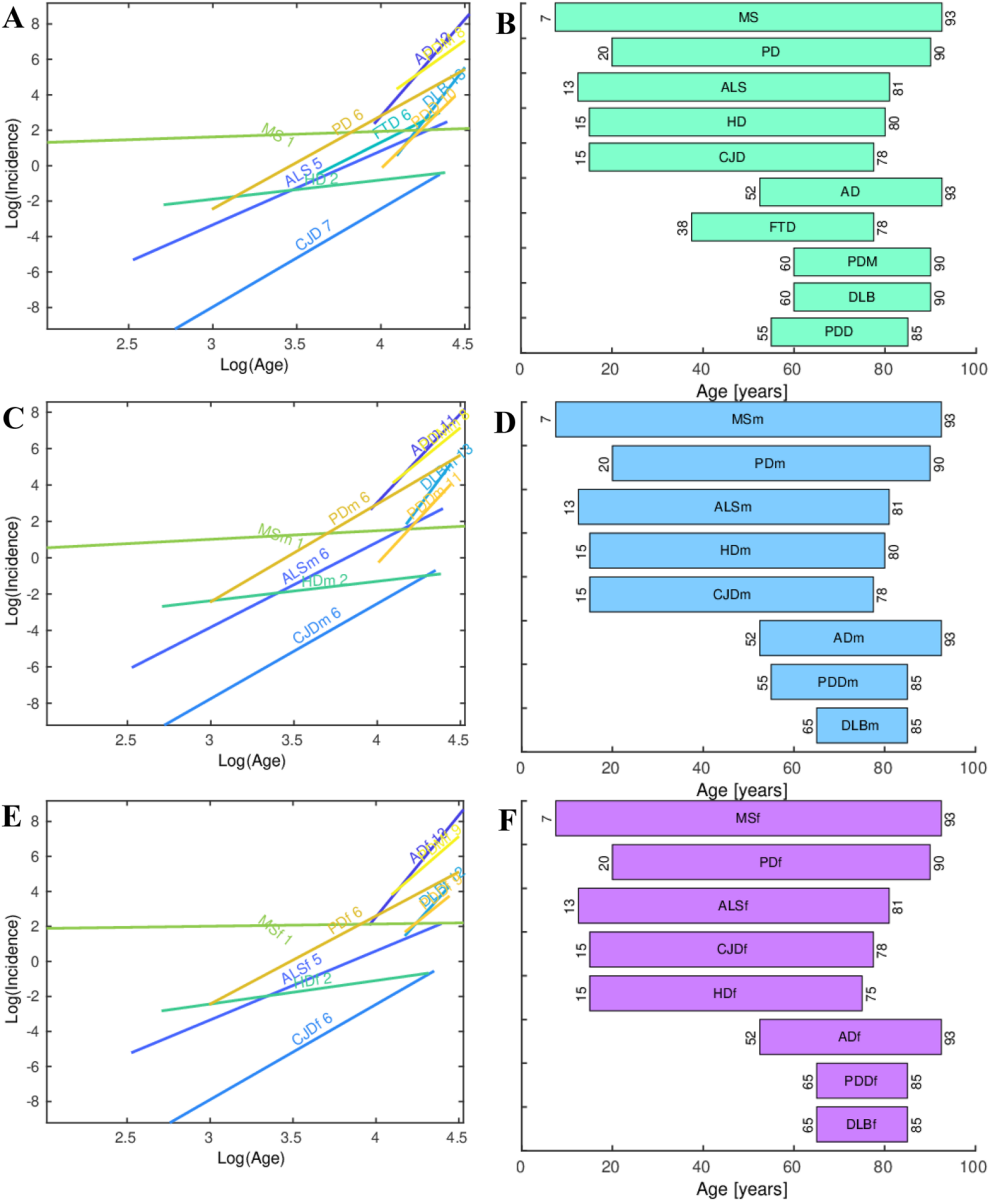
Regression plots of the log_10_ incidence versus the log_10_ age for each ND. **A** All data. **C** Male. **E** Female. The number of steps estimated for each specific ND using the multistep model and used in the genealogy tree model is shown next to the name of each ND. Age-range intervals of the onset of each ND. **B** All data. **D** Male. **F** Female

**Fig. 4:**
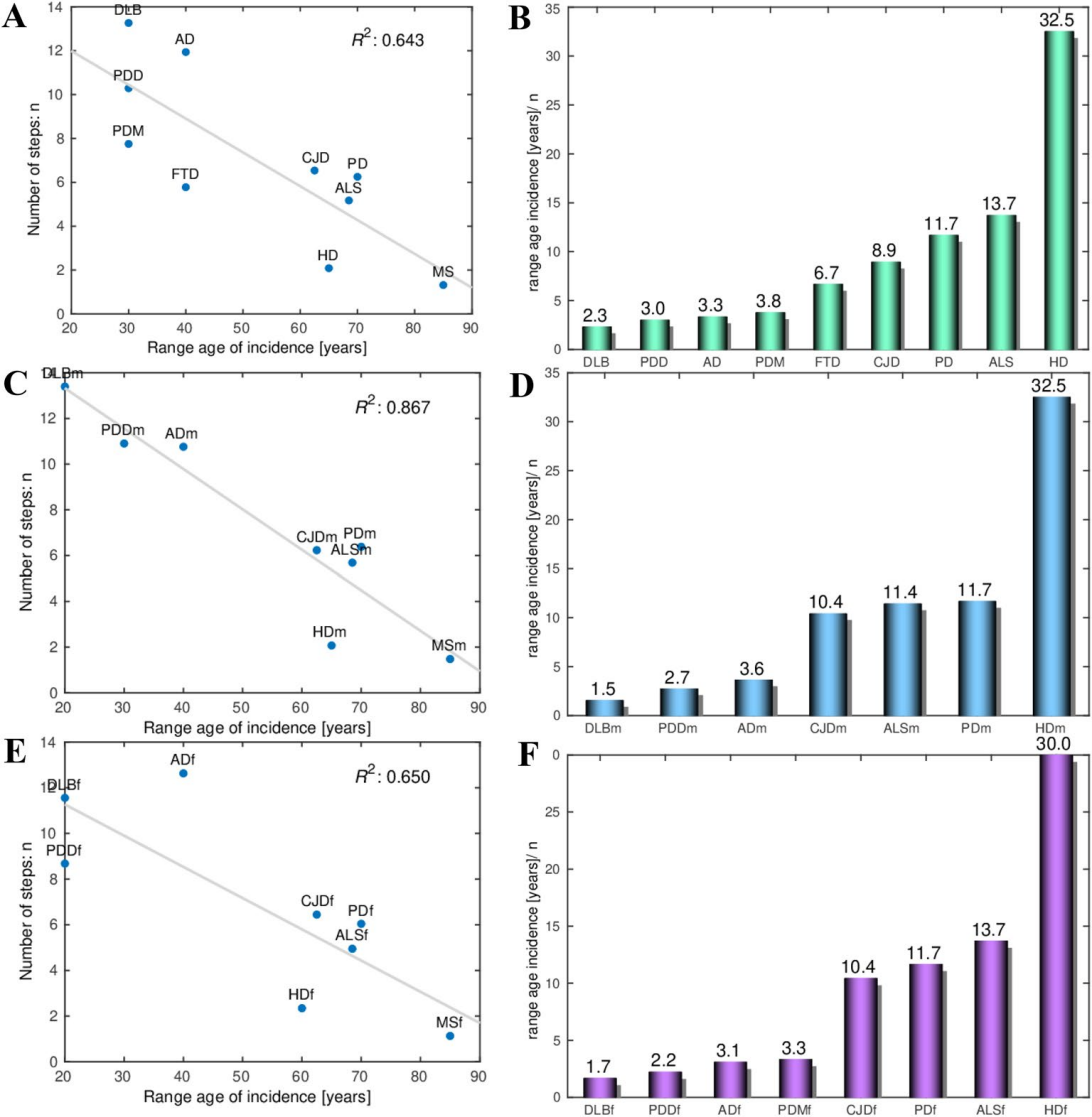
The number of steps *n* versus the range of the age of incidence of each ND. *R*^2^ is the regression coefficient of the fit to a regression*.* **A** All data. **B** Male. **C** Female. Bar plot of the range of the age of incidence divided by the number of steps *n* for each ND. **B** All data. **D** Male. **F** Female

**Fig. 5:**
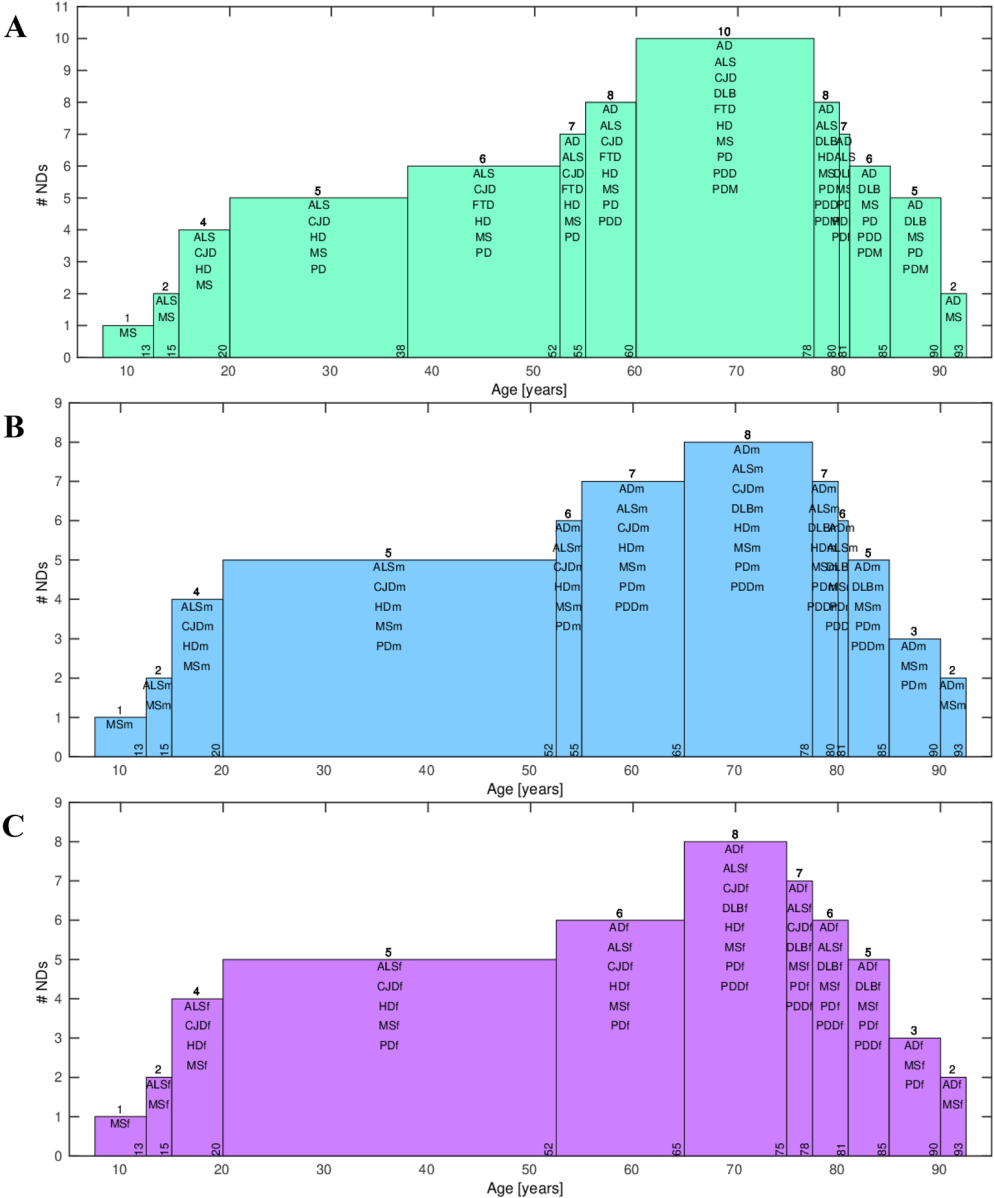
Ranges of age shared by the different NDs. **A** All data. **B** Male. **C** Female. The number on the top of each bar shows the number of NDs having an onset at the age range

Here we revisit the common multistep model of the NDs to account for the relationship between the age range of incidence and the number of steps of the specific diseases. First, we fitted a linear regression model for the slope *m* versus the age of incidence to each of the 10 NDs we have included in our analysis, and estimated the number of steps necessary for its onset (Fig. 3A, C, E). We made analysis of the combined data to include the FTD, for which there is not enough sex-annotated incidence-age data. Next, we represented the age range intervals of the onset of each of the NDs (Fig. 3B, D, F), with MS whose incidence-age profile is not linear, having the widest age interval of onset, whereas AD, DLB and PDD have the shortest ones. Then, we fitted a linear regression model for the number of steps *n* versus the range of the age of incidence of all the NDs. Importantly, the regression coefficients of 0.64, 0.87, 0.65 for all, male and female, show that there is a good correlation between the range age of incidence and the number of steps required to trigger each ND (Fig. 4A, C, E). The number of steps *n* required to trigger a ND is inversely proportional to the range of incidence of the ND. In other words, higher range of the age of incidence of a ND corresponds to smaller number of steps *n* required to trigger the ND. Finally, we calculated the average number of years necessary for a step to occur for each ND (Fig. 4B, D, F). The grouping of the NDs according to the number of years per step is especially well pronounced in the male data, with DLBm, PDDm and ADm forming a group with a mean of 2.6 years/step; CJDm, ALSm and PDm with a mean of 11.7 years/step, and HDm with 32.5 years/step. This is valid also, to a slightly lesser extent, for the combined and female data. We have checked which are the ranges of age shared by the different NDs (Fig. 5). The age interval with most NDs is the one that spans from 60 to 78 years with all the 10 NDs having incidence in that age-range interval (Fig. 5A). After this peak, the number of NDs starts to decrease and only AD and MS remain to occur in the latest age range.

### The trunk of the extended genealogy tree has three sections

**Fig. 6:**
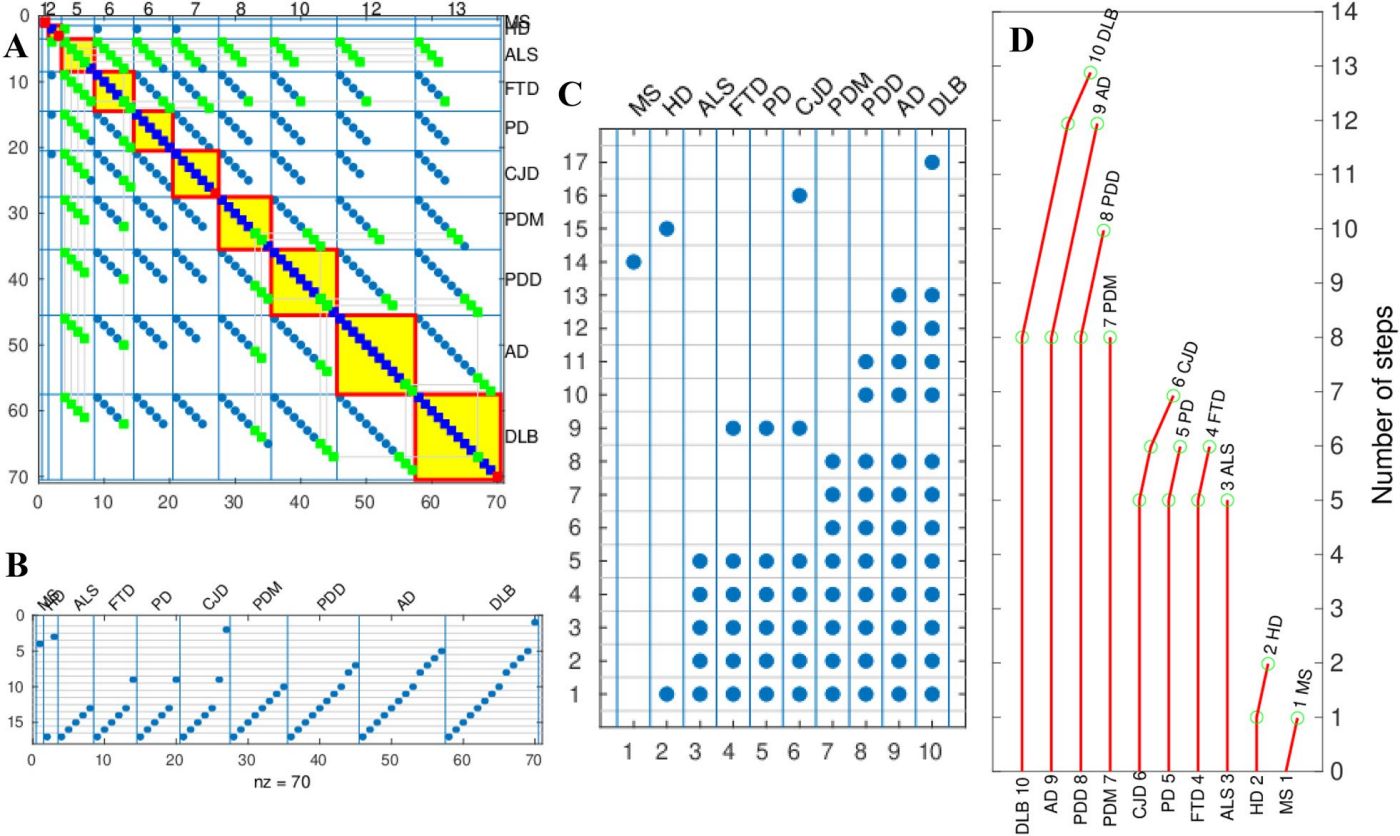
Calculation of the genealogy tree of the NDs for all data, male, female and non-annotated for sex data. **A** Spy of the matrix of adjacency matrix *A* that stores all possible combinations in which a step might be shared by a set of diseases [5] before sorting. Blue squares mark the steps in general. The green squares mark promoters of common steps. The promoters of common steps are marked only in the first disease where the step is found to be common but not in the remaining diseases sharing this common step. Promoter is the first common step found. Red squares mark the non-common steps. The red-bordered yellow squares mark the spy of the matrices used to position each ND on the tree of the genealogy of NDs. **B** Spy matrix of the steps in ordinates marked as blue points distributed across all possible steps shared by all NDs in abscissas. **C** Spy matrix of the steps in ordinates marked as blue points distributed across steps of each ND in abscissas. **D** Number of common and non-common steps calculated for each ND. The number of common steps of each ND is proportional to the length of the vertical red lines and the number of non-common steps is proportional to the length of the red deflection lines to the right. The number of steps is represented in the ordinates. Each ND has an associated ND index given in the spy matrix of **C**. The green circles mark the position of the steps that are non-common

**Fig. 7:**
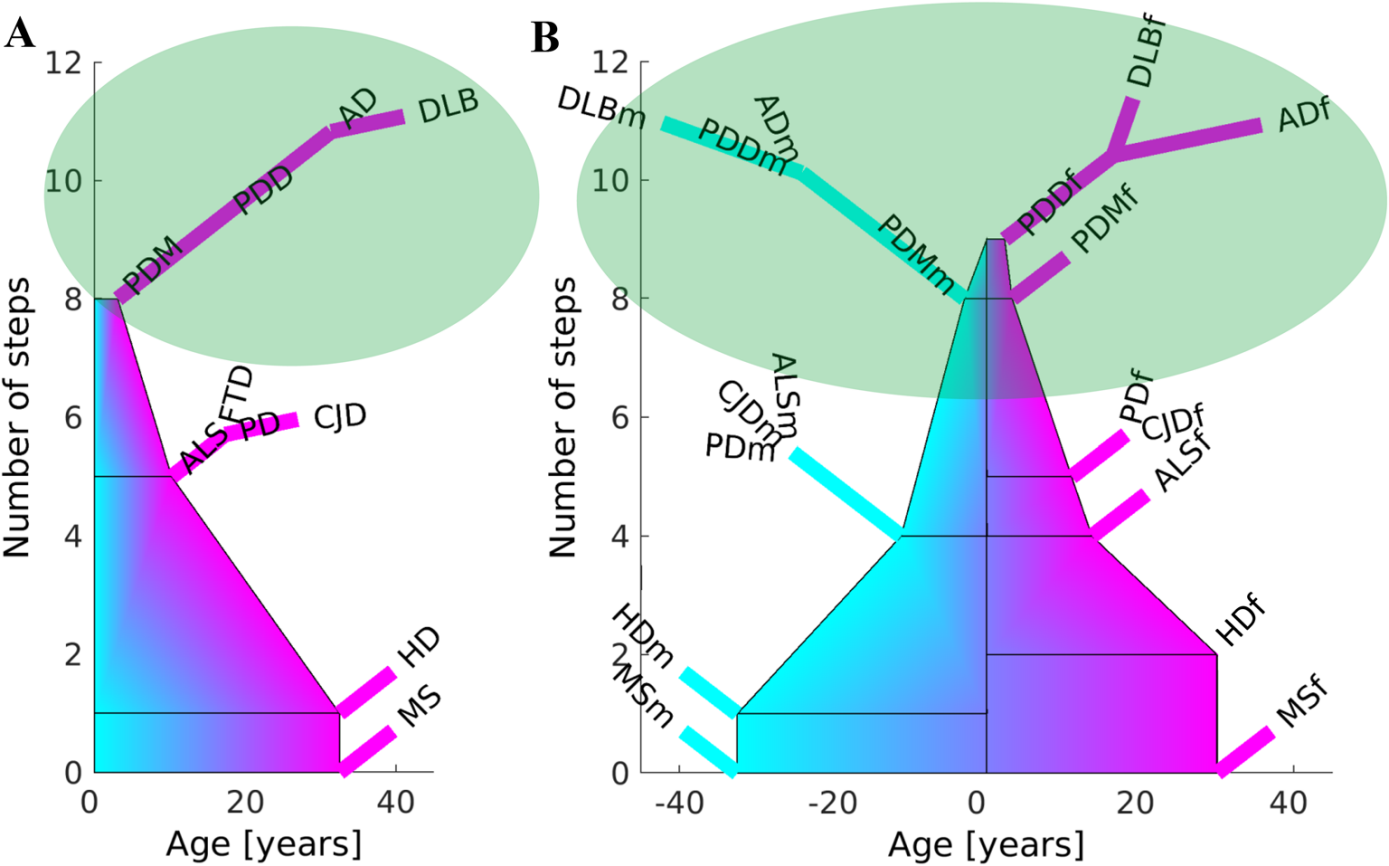
Extended genealogy tree of the NDs based on incidence-age epidemiological data. **A** For all data. **B** Split by sex, male m and female f. The ordinate, or the height of the tree, shows the number of steps of the NDs, with the number of common steps represented by the height of the trunk, and the total number of steps for each ND equal to the number of the abscissa unit segments to the end of the branch of the specific ND. The abscissa or the width of the trunk at the point from each the disease-specific steps for the NDs brunch out, shows the average number of years required for the trigger of one step

First, we recalculated the model of the genealogy tree based on all data [5] excluding only 7 datasets of pooled data. Four out of the datasets have male and female counterpart datasets included in the model, while three had only not annotated for sex, very small by area and population data. The new model based on all data has readjusted the number of shared steps for AD with the other NDs (Fig. 6). We incorporated the mean number of years required for each ND step to occur to the trunk of our genealogy tree for all the data, and for the male and female data. The trees for all data, and separated for male and female are presented in Fig. 7A, B, respectively. The number of common steps among the NDs are represented by the height of the trunk of the tree, while the number of years/step for a ND are represented by the width of the trunk at the point from each the specific steps for the NDs brunch out. The disease-specific steps branch out from the trunk of the tree.

## Discussion

Our extended genealogy tree model suggests the existence of three categories of NDs based on the number of years to pass each step of the disease. The long-time step diseases like the HD, the medium-time step diseases such as ALS, FTD, CJD and PD, and the short-time step NDs such as AD, DLB, PDD and PDM. These three types of steps could provide a hint to impulse the discovery of the mechanisms that trigger such steps. Interestingly, whereas PD belongs to the groups of NDs with middle range of number of steps and middle number of years per step, PDD and PDM are part of the group of NDs with high number of steps and little number of years per step, pointing to additional mechanisms required to pass from PD to PDD and PDM. Any factor associated with the onset of a specific ND disease may be relevant for understanding disease pathogenesis. Modeling disease incidence with age demonstrates some insight into relevant risk factors involved in the disease onset; however, these factors are difficult to identify and the disease outcome can differ if competing risks are considered [9]. It is still unknown whether the neurodegenerative disorders follow a unifying mechanism for disease initiation and propagation, and it might be too soon to decide whether all these disorders should be treated in a similar fashion [20]. Dynamic models like our genealogy tree of the NDs based on incidence-age data might help determine whether there are common mechanisms for the different neurodegenerative disorders, which in turn might aid in our understanding of disease mechanisms and move drug development forward.

## Conclusion

We extended the general multistep model of the most common NDs based on incidence-age epidemiological data in the context of the number of years required for a single step for each ND to occur, and presented it as a new revised genealogy tree of the NDs. The new tree shows three groups of NDs clustered together along the tree trunk according to both the number of steps necessary for their onset, and the years per step. The integration of all the available ND incidence-age epidemiological data and joint models of these, and the inclusion of other NDs whose log(incidence)-log(age) data follows a multistep model can bring new insights into the neurodegenerative processes and identify their stages.

## Data Availability

All data used is publicly available.
